# Modified Valsalva maneuver after burr-hole drainage of chronic subdural hematomas: A single-center cohort study

**DOI:** 10.3389/fneur.2022.1069708

**Published:** 2023-01-30

**Authors:** Lang Zeng, Jiasheng Yu, Rudong Chen, Hongkuan Yang, Hua Li, Lingcheng Zeng, Junhong Wang, Weidong Xu, Shengqi Hu, Kun Chen

**Affiliations:** Department of Neurosurgery, Tongji Hospital, Tongji Medical College, Huazhong University of Science and Technology, Wuhan, Hubei, China

**Keywords:** chronic subdural hematoma (cSDH), modified Valsalva maneuver (MVM), recurrence, predictors, favorable prognosis

## Abstract

**Background:**

Previous studies on the management of chronic subdural hematoma (cSDH) mainly focused on the risk of postoperative recurrence and measures to prevent it. In this study, we propose the use of a non-invasive postoperative treatment method, the modified Valsalva maneuver (MVM), as a means of reducing the recurrence of cSDH. This study aims to clarify the effects of MVM on functional outcomes and recurrence rates.

**Methods:**

A prospective study was conducted at the Department of Neurosurgery, Tongji Hospital, Tongji Medical College, Huazhong University of Science and Technology from November 2016 to December 2020. The study included 285 adult patients who underwent burr-hole drainage for the treatment of cSDH and received subdural drains. These patients were divided into two groups: the MVM group (*n* = 117) and the control group (*n* = 98). In the MVM group, patients received treatment with a customized MVM device for at least 10 times per hour, 12 h per day. The study's primary endpoint was the recurrence rate of SDH, while functional outcomes and morbidity 3 months after surgery were the secondary outcomes.

**Results:**

In the current study, 9 out of 117 patients (7.7%) in the MVM group experienced a recurrence of SDH, while 19 out of 98 patients (19.4%, *p* < 0.05) in the HC group experienced a recurrence of SDH. Additionally, the infection rate of diseases such as pneumonia (1.7%) was significantly lower in the MVM group compared to the HC group (9.2%, *p* < 0.001, odds ratio (OR = 0.1). After 3 months of the surgery, 109 out of 117 patients (93.2%) in the MVM group achieved a favorable prognosis, compared to 80 out of 98 patients (81.6%) in the HC group (*p* = 0.008, with an OR of 2.9). Additionally, infection rate (with an OR of 0.2) and age (with an OR of 0.9) are independent predictors of a favorable prognosis at the follow-up stage.

**Conclusions:**

The use of MVM in the postoperative management of cSDHs has been shown to be safe and effective, resulting in reduced rates of cSDH recurrence and infection following burr-hole drainage. These findings suggest that MVM treatment may lead to a more favorable prognosis at the follow-up stage.

## Introduction

Chronic subdural hematomas (cSDHs) are the abnormal aggregation of encapsulated, liquefied SDHs and may cause brain compression and other neurological deficits. In recent years, cSDHs have exhibited a continuously increasing incidence rate due to the wide application of anticoagulant regimens and the increasingly aging population (1–4). Despite being a common condition, the lack of class I evidence for the treatment of cSDH has led to inconsistencies in surgical and postoperative management in clinical practice. This has made it difficult for clinicians to make informed decisions about the best course of treatment for their patients with cSDH (5, 6).

The principal surgical approaches for cSDH include craniotomies, twist drill craniotomies, and burr-hole drainage. Other procedures are undertaken much more rarely. Weigl et al. (7) found that burr-hole drainage is likely the most effective treatment, with a recurrence rate of 12.1%. Peng and Zhu (8) elaborated that the recurrence rate was significantly lower and that time-to-recurrence was longer in the drain group than those in the no-drain group (*p* = 0.0247). Burr-hole drainage is currently the most commonly used method for the treatment of cSDHs worldwide (1, 7, 9–12).

Notably, the recurrence rates of cSDH after the first treatment were 5–30% (12–14). Edlmann et al. (15) conducted a randomized controlled study of 26 trials examining the use of medical treatment, including tranexamic acid and steroids, as well as middle meningeal artery embolization, for the management of cSDH. Nevertheless, the effects of postoperative management on cSDH recurrence rates remain unclear.

It is generally believed that the postoperative hematoma volume can contribute to the inflammatory process and serve as an indicator of cSDH recurrence (13, 14, 16–19). Indeed, maximum hematoma evacuation is supposed to be a key objective of cSDH treatment. As a result, the modified Valsalva maneuver (MVM) has been involved in postoperative management at our institution for a decade. The MVM is based on the idea of increasing intracranial pressure and expanding the brain, which helps to facilitate the drainage of blood products.

This study aims to investigate the effects of MVM supervision after burr-hole drainage on cSDH recurrence so as to improve the most effective postoperative management methods.

## Materials and methods

### Study design

From November 2016 to December 2020, all patients (*N* = 326) with cSDH who received surgical treatment at our institution were enrolled in this study. Herein, patients with acute-on-cSDH, subacute SDH, and acute SDH, and patients who were not able to provide consent and/or had no subdural drain, were excluded. Meanwhile, all cases with a postoperative Glasgow Coma Scale (GCS) below 15 were excluded, which resulted in some selection bias. Consequently, this study was carried out in a “semi-randomized” fashion. A total of 215 patients (171 men and 44 women aged 56–90 years) were included after they met the inclusion criteria. They were divided into two groups based on the admission date: those in the experimental group were admitted on dates of even numbers, while those in the control group were accepted on days of odd numbers. As a result, the two groups were effectively matched. They all underwent surgical burr-hole drainage at the Department of Neurosurgery, Tongji Hospital, and Tongji Medical College. This study received approval from the Ethics Committee of Tongji Hospital, and all participants signed the informed consent form voluntarily with the review of the same committee.

### Surgical approach

After inducing general anesthesia, the patients were positioned supine on a horseshoe headrest, and two burr holes (size = 1.4 cm, spacing = 7 cm) were drilled over the maximum width of the hematoma. A cruciate incision was made to open the dura mater, which was then coagulated using bipolar diathermy. Thereafter, a flexible silicon drain from Suzhou Xinda Medical Equipment Co., Ltd., Jiangsu, China, with dimensions of 30 cm in length and 4.7 mm in external diameter, was inserted into the subdural space (SDS) *through* burr-holes located above the large section of the subdural cavity. The drain was tunneled a minimum of 5 cm from the incision without disrupting any other subdural membranes. The burr holes were irrigated with Ringer's lactate saline at a temperature of 36°C using the drains. The bilateral hematoma was treated as a single case, and the identical treatment was applied to both sides. Afterward, the SDS was filled with saline, and the scalp was closed in two layers. The drains were connected to soft collection bags placed at the bedside for 24–72 h. The drainage system was maintained at a height equal to that of the external auditory canal.

### Procedures

Clinical status and essential medical history, including the use of anticoagulants and antiplatelet agents, were assessed upon admission. Preoperatively, all patients underwent brain CT scans and MRI scans. Won et al. verified the high accuracy of the ABC/2 volume formula for volume analysis of subdural hematoma (20). Hence, the ABC/2 volume formula was employed in this study. The maximal deviation of the midline structures was measured at the foramen of Monroe level to enable the calculation of the midline shift. Additionally, postoperative cranial CT imaging was routinely performed on the day the drainage tube was removed. If the patient's health did not improve or became worse after treatment, a reoperation by craniotomy was performed.

**Figure 1:**
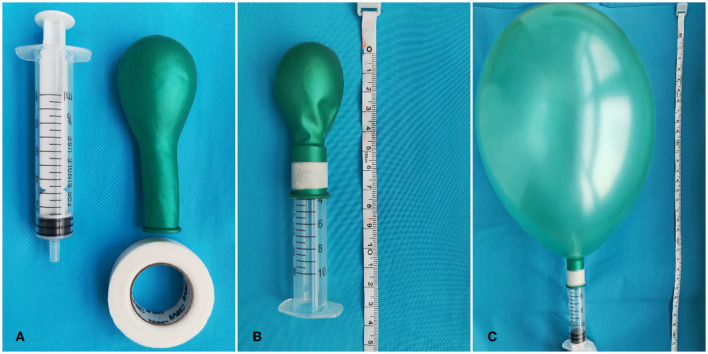
Production of the MVM device. **(A)** Materials: 10 ml syringe (Hongda Medical Equipment Group Co., Ltd., Jiangxi, China), one medium-size balloon (Piao Fa Industrial Co., Ltd., Shenzhen, China), and leucoplast [Minnesota Mining Manufacturing Medical Equipment (Shanghai) Co., Ltd.]. **(B)** The balloon is circled around the syringe and fixed by leucoplast. **(C)** After blowing into the syringe, the balloon will be in full tension. We defined this procedure as an effective Valsalva maneuver.

Figure 1 illustrates the production of the MVM device. After fully awakening from anesthesia, the subjects in the MVM group followed a stepwise protocol under the supervision of experienced physicians. While MVM was being performed, the patients were strictly kept in a semi-sitting position and asked to inhale deeply before blowing into a syringe to inflate an attached latex balloon. In an effective maneuver, the subjects were asked to hold their breath for 10 s once the balloon reached a diameter of 15–20 cm and then release the air. Based on our prior experience, this procedure was carried out safely at least 10 times per hour and for 12 h each day for 3 days.

After surgery, a 3-month follow-up was conducted for all subjects. At both the discharge and follow-up stages, the neurological outcomes were assessed based on the modified Rankin Scale (mRS), and the hematomas were investigated using CT scanning.

The recurrence rate would be the primary outcome. All recurring events in our study occurred after the drainage was performed. Reoperation is often performed because of the significant reappearance of hematomas after previous clearance, with or without recurrent hematoma-related symptoms. In this study, all reoperations were performed within 3 months of the initial treatment. Notably, the need for reoperation was assessed without distinguishing between the two groups. Additionally, functional outcomes and morbidity 3 months after surgery served as the secondary outcomes.

### Statistical analysis

The Kolmogorov–Smirnow test was employed to determine normality. The *t*-test and the Mann–Whitney U test were applied to the data that followed a normal distribution and those that did not, respectively. The categorical variables were compared using the chi-square test. Additionally, the interquartile range and median of the numerical data were calculated. A logistic regression was performed to clarify the effects of MVM and the other independent variables mentioned previously on the favorable outcome and the recurrence rate (mRS 0–2) at discharge and follow-up. The OR with a 95% confidence interval (CI) of variables in this study was calculated. *p* < 0.05 denote statistical significance. SPSS software for Windows (Chinese Version 22.0) was employed for data analysis.

### Statement of data availability

We confirmed that all the data supporting the conclusions of this study are available in the manuscript and/or the supplementary material.

## Results

**Figure 2:**
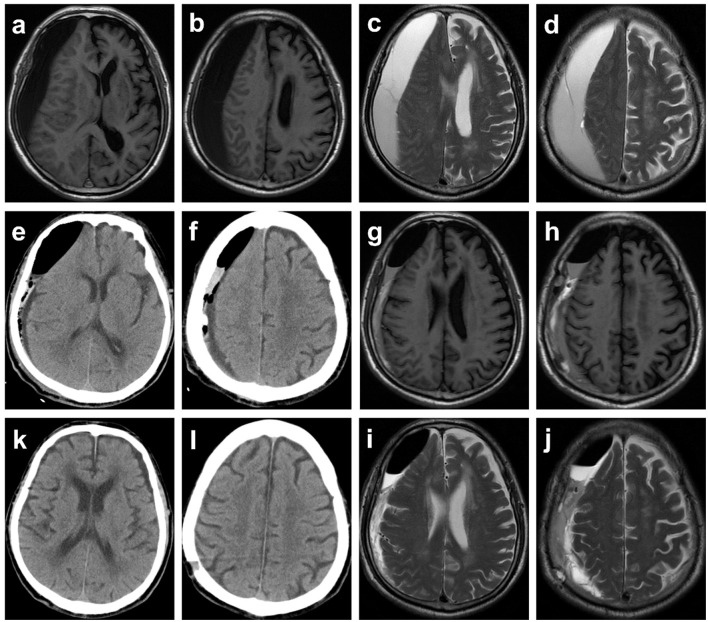
Typical case 1. Images obtained from an 81-year-old man with chronic subdural hematoma (cSDH) from the MVM group. **(a–d)** Preoperative T1-weighted and T2-weighted MR images. **(e, f)** CT reexamined the day after surgery demonstrating that the midline of the brain returned to normal, yet part of blood products and air remained. The patient was asked to perform the modified Valsalva maneuver. **(g–j)** Postoperative (a week) T1-weighted and T2-weighted MR images. **(k, l)** A follow-up CT scan obtained 1 month later demonstrated resolution of cSDH, and symptoms of headache and hemiparesis were significantly relieved.

**Figure 3:**
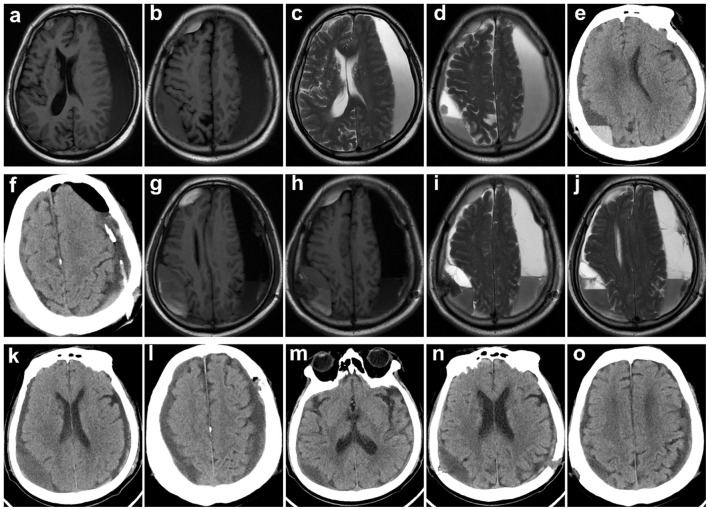
Typical case 2. Images obtained from a 79-year-old man with bilateral cSDH from the HC group. **(a–d)** Preoperative T1-weighted and T2-weighted MR images. **(e, f)** CT reexamined the day after surgery showing that the midline of the brain returned to normal (symptoms of hemiparesis and speech arrest were relieved), yet part of blood products and air remained. **(g–j)** MRI reexamination was given on the fifth day after the operation, because of impaired consciousness and the symptoms of hemiparesis and speech arrest reappeared. **(k, l)** Reoperation of burr-hole drainage was conducted. **(m–o)** A follow-up CT scan obtained 1 month later demonstrated resolution of cSDH, and symptoms were significantly relieved.

**Figure 4:**
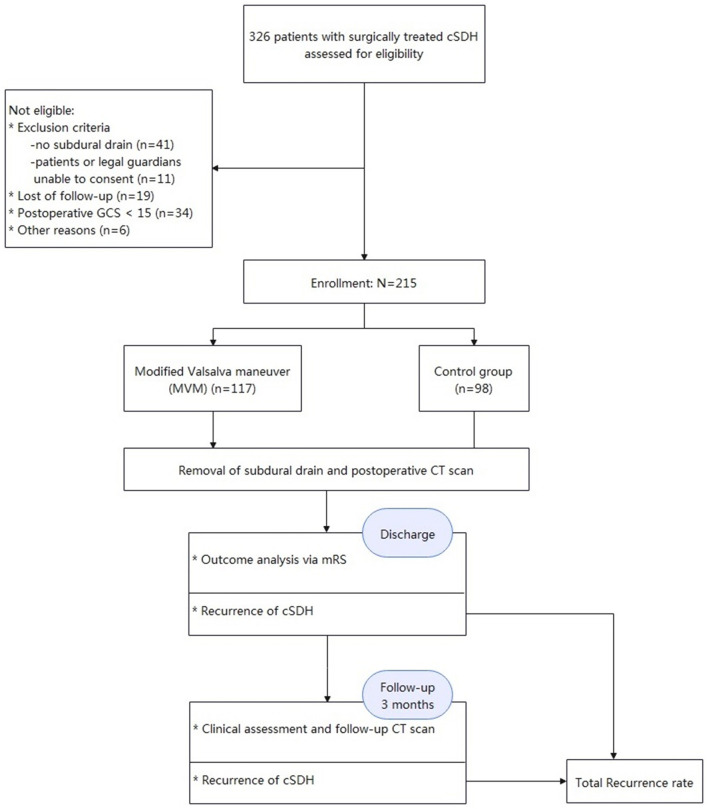
The overall design of this study.

Figures 2, 3 show typical cases. Figure 4 illustrates the overall design of this study. In this study, 326 patients with cSDH were surgically treated, while 111 of them were excluded. Eventually, 215 subjects were enrolled. Among them, 117 and 98 were categorized into the MVM and HC groups, respectively. Table 1 summarizes the clinical and demographic characteristics of all the subjects enrolled. No radiological or clinical differences (e.g., uni-/bilateral hematoma, hematoma side, midline shift, hematoma volume, complications, or GCS on admission) were observed among the two groups, resulting in a pair that is well matched. In most cases, the subdural drain placement lasted for 2 days (91.2%).

**Table 1:** ^*^Demographic data (*N* = 215).

	**MVM group (*n* = 117)**	**HC group (*n* = 98)**	**p-value**
Age^#^ (years)	75 ± 8.7 (min 56–max 90)	72 ± 7.5 (min 59–max 87)	0.43
Sex (male)	94 (80.3%)	77 (78.6%)	0.22
**Medical history**			
Hypertension	62 (53.0%)	54 (55.1%)	0.31
Diabetes mellitus type II	19 (16.2%)	15 (15.3%)	0.17
Atrial fibrillation	11 (9.4%)	8 (8.2%)	0.85
Cardiovascular disease	25 (21.4%)	20 (20.4%)	0.41
Coronary disease	23 (19.7%)	19 (19.4%)	0.68
Respiratory disease	27 (23.1%)	21 (21.4%)	0.36
Renal disease	8 (6.8%)	7 (7.1%)	0.28
Ischemic stroke (including TIA)	17 (14.5%)	14 (14.3%)	0.20
Hematologic disease	7 (6.0%)	6 (6.1%)	0.44
Oncology	11 (9.4%)	7 (7.1%)	0.69
**Drug history**			
Anticoagulation or Antiplatelet	56 (47.9%)	46 (46.9%)	0.70
**GCS on admission**			
13–15	107 (91.5%)	89 (90.8%)	0.30
7–12	10 (8.5%)	9 (9.2%)	0.30
3–6	0 (0.0%)	0 (0.0%)	1.00
**CT**			
Volume, median (IQR)	119.4 (81.5–152.4)	112.1 (69.3–143.5)	0.92
Midline shift, median (IQR)	6.6 (4.2–10.1)	6.2 (4.5–9.6)	0.58
**Side**			0.60
Left	41 (35.0%)	35 (35.7%)	
Right	42 (35.9%)	33 (33.7%)	
Both	34 (29.1%)	30 (30.6%)	
**Density**			
Hypodense	58 (49.6%)	46 (46.9%)	0.11
Isodense	42 (35.9%)	34 (34.7%)	0.52
Hyperdense	17 (14.5%)	18 (18.4%)	0.47
**Operation**			
Burr-hole drainage	117 (100.0%)	98 (100.0%)	1.00
Drain	117 (100.0%)	98 (100.0%)	1.00
< 2 days	4 (3.4%)	3 (3.1%)	0.18
=2 days	106 (90.6%)	90 (91.8%)	0.14
>2 days	7 (6.0%)	5 (5.1%)	0.44

^*^Chi-square test was used for parametric statistical analysis. Mann-Whitney U-test was used for non-parametric statistical analysis.

IQR, interquartile range; TIA, transient ischemic attack; GCS, Glasgow coma scale.

^#^Mean ± standard deviation.

Table 2 illustrates the symptoms on admission. As observed, the most common symptoms included headache (41.9%), gait impairment (35.3%), speech arrest (34.4%), impaired consciousness (26.0%), hemiparesis (24.7%), and nausea/vomiting (14.4%). Most subjects (37.67%) had two symptoms, while 27.44% and 23.26% of the subjects had one and three symptoms, respectively; five subjects (2.33%) had over five more symptoms on admission.

**Table 2:** Symptoms on admission of cSDH patients.

Headache	90 (41.9%)
Gait impairment	76 (35.3%)
Hemiparesis	53 (24.7%)
Speech arrest	74 (34.4%)
Nausea/vomiting	31 (14.4%)
Impaired conciousness	56 (26.0%)
Seizure	11 (5.1%)
Sensory deficit	10 (4.7%)
Syncope	7 (3.3%)
Incontinence	2 (0.9%)
**Cumulative number of symptoms on admission (*****N*** = **215)**	
Asymptomatic	10 (4.65%)
1 symptom	59 (27.44%)
2 symptoms	81 (37.67%)
3 symptoms	50 (23.26%)
4 symptoms	10 (4.65%)
5 symptoms or more	5 (2.33%)

The hematoma recurrence of the MVM group was significantly reduced compared with the HC group [7.7% vs. 19.4%; *p* = 0.02; with OR of 0.5 95% CI = (0.2–1.0)] 3 months after surgery (Table 3). Herein, possible confounding variables, including uni- or bilateral hematoma, age, clinical course infection, GCS on admission, and neurological deficit (aphasia, gait impairment, or paralysis), were adjusted. A logistic regression analysis demonstrated that MVM is a single significant parameter related to reduced recurrence rate [OR = 0.5, 95% CI = (0.2–1.0)] (Table 4).

**Table 3:** Primary and secondary outcome analysis in MVM group and HC group (*N* = 215).

	**MVM group** **(*n* = 117)**	**HC group** **(*n* = 98)**	**p-value**	**OR (Cl 95%)**
Recurrence within 3 months	9 (7.7%)	19 (19.4%)	0.02	0.5 (0.2–1.0)
**Favorable outcome (mRS 0–2)**				
At discharge	100 (85.5%)	76 (77.6%)	0.004	2.8 (1.4–6.1)
At 3 months	109 (93.2%)	80 (81.6%)	0.008	2.9 (1.3–7.1)
**Mortality rate**				
At discharge	0 (0.0%)	0 (0.0%)	1.00	
At 3 months	0 (0.0%)	1 (1.0%)	0.28	
Morbidity	8 (6.8%)	18 (18.4%)	< 0.001	0.1 (0.0–0.4)
Infection^#^	2 (1.7%)	9 (9.2%)	< 0.001	0.1 (0.0–0.6)
Acute subdural hematoma	4 (3.4%)	6 (6.1%)	0.47	
Epidural hematoma	1 (0.9%)	2 (2.0%)	0.48	
Intracerebral hematoma	1 (0.9%)	1 (1.0%)	1.00	
Hospital stay, median (IQR)	10 (8–12)	9 (8–12)	0.96	

T-test and Fisher exact test were used for outcome analysis.

OR, odds ratio; CI, confidence interval; mRS, modified Rankin Scale; IQR, interquartile range.

^#^Infection includes pneumonia, urinary tract infection and intracranial infection. Two patients in the MVM group were both diagnosed with pneumonia. In the HC group, 5 patients were considered pneumonia, 3 patients got urinary tract infections, and 1 patient was diagnosed with intracranial infection.

**Table 4:** Adjusted logistic regression outcome analysis by variables with potential influence on relation between MVM and recurrence/outcome at discharge as well as at follow-up.

	**Recurrence rate**		**Favorable outcome at discharge**		**Favorable outcome at follow-up**	
	**p-value**	**OR** **(Cl 95%)**	**p-value**	**OR** **(Cl 95%)**	**p-value**	**OR** **(Cl 95%)**
Age	0.64	1.0 (1.0–1.0)	0.001	0.9 (0.8–1.1)	0.002	0.9 (0.8–1.1)
GCS at admission	0.61	1.1 (0.8–1.6)	0.005	1.5 (1.2–2.3)	0.84	1.0 (0.8–1.4)
Neurological deficit	0.25	1.7 (0.8–3.2)	0.57	1.3 (0.5–3.1)	0.90	1.1 (0.4–2.7)
Infection	0.86	1.2 (0.3–4.4)	0.001	0.0 (0.0–0.2)	0.03	0.2 (0.0–0.8)
Coagulopathy or anticoagulation/antiplatelet	0.20	1.6 (0.7–3.5)	0.20	0.7 (0.3–1.7)	0.25	0.6 (0.2–1.5)
Unilateral hematoma	0.21	0.7 (0.5–1.2)	0.21	1.2 (0.7–2.0)	0.15	1.5 (0.9–2.6)
Modified Valsalva maneuver	0.05	0.5 (0.2–1.0)	0.18	1.8 (0.8–4.1)	0.10	2.2 (0.9–5.7)

At the discharge stage, a favorable outcome was observed in 176 (81.9%) of the subjects. Among them, 100 (85.5%) were in the MVM group, and 76 (77.6%) were in the HC group [*p* = 0.004; with an OR of 2.8, 95% CI = (1.4–6.1)]. Three months after the surgery, the probability of favorable outcomes in the MVM group was significantly improved compared with the HC group [*p* = 0.008; OR = 2.9, 95% CI = (1.3–7.1)]. The median duration of hospitalization was 10 days (IQR 8–12), and the two groups had no significant difference in this parameter.

The logistic regression analysis revealed that some independent predictors, including age [OR = 0.9 (0.8–1.1)], GCS at admission [OR = 1.5 (1.2–2.3)], and infection during hospitalization [OR = 0.0 (0.0–0.2)], are related to the outcomes at the discharge stage. At the follow-up stage, the remaining independent predictors included infection (OR = 0.2) and age (OR = 0.9).

Regarding the non-invasive nature and safety of the MVM, neither hemodynamic instability nor syncope episodes were observed in this study. Meanwhile, intracerebral hematoma (*n* = 2), epidural hematoma (*n* = 3), and acute subdural hematoma (*n* = 10) were detected in both groups. Additionally, the infection rates of the MVM group (1.7%) were significantly lower than those of the HC group (9.2%) [*p* < 0.001; with an OR of 0.1 (0.0–0.6)] (Table 3).

## Discussion

Few studies on the effects of MVM on chronic subdural hematoma have been reported to date. The results of this study demonstrated that MVM is an effective measure to reduce SDH recurrence. Meanwhile, compared with the HC group, the MVM group exhibited improved neurological outcomes and reduced infections at the follow-up stage. MVM-related adverse effects were not observed, further demonstrating the favorable safety of MVM.

Multiple predictors associated with cSDH recurrence after evacuation, including preoperative hematoma volume, age, seizures, anticoagulation/platelet status, postoperative residual hematoma, radiological results reflecting the hematoma type (e.g., laminar type, density), and bilateral cSDH (18, 19, 21–26), have been reported. In an effort to reduce SDH recurrence, different surgical techniques (e.g., the number of burr holes, the position of the burr holes (parietal or frontal), burr-hole or craniotomy, the direction of drainage, intraoperative irrigation, subperiosteal or subdural drainage, and fluid temperature in cases of irrigation) were investigated (27–30). Additionally, endovascular interventions (e.g., occlusion of the middle meningeal artery) and medical treatments using agents (e.g., atorvastatin, dexamethasone) have been applied recently to reduce the recurrence rate of SDH. It was expected that the neuroinflammatory axis could be perturbed by such an intervention (16, 31–33). Indeed, the postoperative residual hematoma triggers a continuous inflammation by a series of inflammatory cells, interleukin, and chemokines (16). Hence, the hematoma evacuation shall be maximized using postoperative regimens and surgical interventions. In this study, MVM could facilitate residual hematoma drainage by a subdural drain that was placed intraoperatively. MVM is a forced expiration against a closed glottis, resulting in increased intrathoracic and intraabdominal pressures (34). For this reason, the residual hematoma is removed, and the intracranial pressure is elevated by brain expansion (35, 36). Notably, postoperative CT scans typically suggest collections of subdural air, which is also a risk factor for SDH recurrence (23, 37). Indeed, these patients would also benefit from MVM, as it can relieve such collections.

Hemodynamic instability is another concern related to MVM, as such maneuvers influence blood pressure and/or heart rate through baroreceptor activation (34). More importantly, a risk of rebleeding is present as the MVM may cause increased intracranial pressure. Despite that, this study did not identify bleeding and/or cardiovascular complications secondary to MVM.

The morbidity in this study identifies additional MVM-associated benefits, including low infection rates for diseases such as pneumonia. This can be attributed to the fact that the MVM positively impacts the lungs by preventing postoperative atelectasis. Boden et al. (38) clarified the effect of physiotherapy and breathing exercises on a considerable number of patients (up to 15%) after abdominal surgery. The results suggested a reduced absolute risk of pulmonary complications such as pneumonia. Additionally, mobilization and respiratory physiotherapy play a key role in the functional status and sequelae of pneumonia (39). For this reason, the MVM group exhibited significantly improved outcomes compared to the HC group at both stages (discharge and follow-up).

Our study showed that after the first treatment, recurrence rates of cSDH in the MVM group and in the HC group were 7.7% and 19.4%, respectively, while that in a recently published TOSCANA study was 23% (40). Meanwhile, the recurrence rate of cases with bilateral cSDH reached 28.7% (41). As mentioned above, several factors significantly influence the recurrence rate of cSDH, while the absence of a common postoperative management strategy between different clinics is another reason. Some studies suggested that radiological follow-up was regularly performed, while there was no follow-up in other studies, and the patients were clinically deteriorating. We performed regular radiological follow-ups on all subjects enrolled.

Numerous preoperative and perioperative therapeutic regimes have been proposed in the literature for reducing the recurrence rate of cSDH. However, postoperative treatment is usually excluded. The results of this study suggest that MVM is a facile, effective, yet safe postoperative treatment that exhibits reduced postoperative recurrent hematoma.

## Limitations

It should be noted that our study is not randomized and may be subject to selection bias. However, the groups were well matched. One challenge in MVM is the need for individual effort, and it is possible that some patients were more motivated to participate in MVM than others. Future studies may focus on addressing motivation for MVM in all parties involved.

## Conclusion

Modified Valsalva maneuver has been demonstrated to be both safe and effective for preventing infections and reducing the recurrence rate of cSDHs following surgery. The outcomes are favorable at the follow-up stage. Its excellent safety profile and reproducibility make it a promising treatment option that can be implemented in both high- and low-resource settings, potentially improving outcomes for neurosurgical patients.

## Data availability statement

The original contributions presented in the study are included in the article/supplementary material, further inquiries can be directed to the corresponding author.

## Ethics statement

The study involving human participants was reviewed and approved by the Ethics Committee of Tongji Hospital. The patients/participants provided their written informed consent to participate in this study.

## Author contributions

Zeng L, M.D and Yu JS, Ph.D contributed to the conception of the study. Chen RD, Yang HK, Li H and Zeng LC performed the experiment. Wang JH and Xu WD contributed significantly to analysis and manuscript preparation. Zeng L performed the data analyses and wrote the manuscript. Hu SQ and Chen K helped perform the analysis with constructive discussions.
